# First genetic detection and characterization of canine parvovirus type 2 (*Carnivore protoparvovirus 1*) in southwestern Ethiopia

**DOI:** 10.1007/s11259-022-10027-4

**Published:** 2022-11-02

**Authors:** Dechassa Tegegne, Girma Tsegaye, Giulia Faustini, Giovanni Franzo

**Affiliations:** 1 School of Veterinary Medicine, Jimma University College of Agriculture and Veterinary Medicine, P.O. Box 307, Jimma, Ethiopia; 2 Department of Animal Medicine, Production and Health (MAPS), University of Padua, Viale Dell’Università 16, 35020 Legnaro, PD Italy

**Keywords:** CPV-2, Ethiopia, Phylogenesis, Molecular epidemiology, Dog

## Abstract

The species *Carnivore protoparvovirus 1* includes viruses, e.g. canine parvovirus (CPV-2) and feline panleukopenia virus (FPV), which are among the most relevant for pets, causing extremely severe clinical signs and high fatality rate in dogs and cats. Moreover, a broad range of wild hosts, including endangered ones, were proven to be susceptible. Currently, no data on CPV-2 molecular epidemiology and strain characterization are available in Ethiopia, also considering the frequent contacts between domestic and wild populations. In the present study, a molecular epidemiology survey was performed on 92 fecal samples collected from domestic (*n* = 84) and stray (*n* = 8) dogs in southwestern Ethiopia in 2021. Approximately, 10% of the samples tested positive and the complete VP2 sequences of 5 strains were obtained, classified within the CPV-2a (*n* = 1) and CPV-2c (*n* = 4) antigenic variants. In most instances, the closest genetic relatives were strains of Chinese origin, which is fully compatible with the intense relationships that have been developing between the two countries, involving human being travels and their pets as well. Considering the clinical relevance of this infection and the risk it poses to local domestic and wild carnivore populations, especially endangered ones, much stronger monitoring and surveillance activity on foreign incoming animals should be performed. More strict constraints on animal introduction, e.g. compulsory vaccination, should also be considered.

**Supplementary Information:**

The online version contains supplementary material available at 10.1007/s11259-022-10027-4.

## Introduction

Canine parvovirus type 2 (CPV-2) has been recently classified, together with feline panleukopenia virus (FPV), mink enteritis virus (MEV) and racoon parvovirus (RaPV), in the species *Carnivore protoparvovirus 1*, genus *Protoparvovirus*, family *Parvoviridae* (Cotmore et al. 2014). It includes non-enveloped viruses with a single-stranded DNA genome of approximately 5 kb (Paul Reed et al. 1988). Two open reading frames encodes for two non-structural proteins (NS1-2), involved in viral replication, and three structural proteins (VP1-3). In particular, the VP2 protein is the main constituent of the viral capsid, is responsible for the viral attachment, contributes to the cell and host tropism, and is the main target of the host immune response (Parrish 2010). Evidence of the association between virulence and viral phylogenesis, reconstructed based on this segment, has been provided (Franzo et al. 2019; Tucciarone et al. 2021). Moreover, because of its remarkable genetic variability, VP2 has been widely sequenced for molecular epidemiology and evolutionary studies (Miranda and Thompson 2016; Tucciarone et al. 2018). In fact, as other ssDNA viruses, carnivore protoparvoviruses are featured by a noteworthy evolutionary rate (Hueffer and Parrish 2003), which facilitate them to promptly adapt to new environments and especially hosts (Shackelton et al. 2005). CPV is assumed to originate from FPV or FPV-like virus adaptation to the canine host through wild intermediate (Franzo et al. 2017; Hoelzer and Parrish 2010; Hueffer and Parrish 2003). Further evolution led to the emergence of different antigenic variants, namely CPV-2a, -2b and -2c, which regained the ability to infect cats and replaced the original CPV-2 variant. The classification of such variants is based on the presence of distinctive amino acids in specific residues of the VP2 protein and does not necessarily reflect the ancestry relationship among strains (Decaro and Buonavoglia 2012; Tucciarone et al. 2018). Besides domestic dogs and cats, FPV, CPV-2 and its antigenic variants have been reported in several wild species (Steinel et al. 2001).

CPV infection can be responsible for severe clinical signs, including vomiting and diarrhoea, in association with anorexia, depression and fever. Fluid and protein losses through the gastrointestinal tract can lead to severe dehydration and hypovolemic shock. Immunosuppression develops as a consequence of the direct viral targeting of the leucocytes and their progenitor cells. Severe systemic inflammatory response syndrome due to bacterial septicemia and/or endotoxin absorption through the damaged intestine is also common, leading to mortality in untreated subjects as high as 91% (Kalli et al. 2010; Otto et al. 1997; Prittie 2004; Schoeman et al. 2013). It is thus clear that CPV-2 circulation represents a threat not only to the health and welfare of domestic animals but also to wild species and the consequences can be especially severe for endangered ones, contributing to their population size decline (Alexander and Appel 1994; Sillero-Zubiri et al. 1996). This threat becomes particularly relevant when contacts between wild and domestic populations, especially unvaccinated ones, are frequent and unconstrained. Such a scenario is commonly encountered in Africa where outdoor-living or stray pets and wild animals, including species of high ecological relevance, may come into contact (Butler et al. 2004; Craft et al. 2017; Hughes and Macdonald 2013).

CPV-2 presence has been documented in some African countries, at high prevalence in diseased dogs (Amrani et al. 2016; Dogonyaro et al. 2013; Figueiredo et al. 2017; Kapiya et al. 2019; Ndiana et al. 2021; Tion et al. 2021; Touihri et al. 2009). However, no information is currently available from the North-Eastern African regions. Based on these premises, an epidemiological study was organized to investigate and characterize the circulation of CPV-2 in Ethiopian domestic and stray dogs.

## Material and methods

### Sample collection

Ninety-two fecal samples were collected from domestic (*n* = 84) and stray (*n* = 8) dogs, both healthy and showing gastrointestinal signs, in the Jimma town, Ethiopia, in 2021. The samples were collected from Hermata, Hermata Mentina, Jimma University Veterinary Clinic and Bacho Bore. Before sample collection, clinical examination was performed by veterinarians that evaluated general appearance, vital signs and performed physical examinations of the dogs. Collection sites and dates, as well as the occurrence of clinical signs, were recorded when possible. Collected samples were refrigerated until processing. Within 24 h after collection 1 g of feces was homogenized in 9 ml of PBS and 125µL of the obtained solution were applied to FTA (Flinders Technology Associates)® Cards (Whatman™) and left to dry for 30 min at room temperature. At the end of the sampling, FTA Cards were delivered to the laboratory of infectious disease of the dept.MAPS, Padua University, Italy, for CPV-2 infection diagnosis and molecular characterization.

### CPV-2 diagnosis and sequencing

A 1 cm·0.3 mm strip of FTA Cards was trimmed, put in a vial containing 500µL of PBS and incubated at room temperature for 1 h while continuously vortexing. 100 µL of the supernatant were collected and extracted using the kit Viral DNA/RNA (A&A Biotechnology) according to manufacture instruction. An exogenous internal control, provided by the QuantiNova Pathogen + IC kit (Qiagen), was added before extraction. The viral detection was performed using the same kit at the following conditions: 1X QuantiNova Pathogen Master Mix, 0.8 µM of forward and reverse primers (CPV-F: 5’- AAACAGGAATTAACTATACTAATATATTTA-3’ and CPV-R: 5’- AAATTTGACCATTTGGATAAACT-3’), and 0.25 µM of probe (FAM-TGGTCCTTTAACTGCATTAAATAATGTACC -BHQ1). 1X QuantiNova IC Probe Assay was also included in each reaction. Biology grade water was added up to a final value of 10 µl. The following thermal protocol was used: 2 min at 95 °C followed by 45 cycles at 95 °C for 5 s and 60 °C for 30 s. Fluorescence was acquired at the end of the extension phase.

Complete VP2 sequencing was attempted on all positive samples using the protocol described by Tucciarone et al. (2018). Sanger sequencing was performed at Macrogen Europe (Amsterdam, The Netherlands).

### Sequencing analysis

Chromatogram quality was evaluated using FinchTV (http://www.geospiza.com) and consensus sequences were assembled with ChromasPro (ChromasPro Version 2.0.0, Technelysium Pty Ltd). The obtained sequences were compared to the complete dataset of CPV-2 and FPV VP2 sequences downloaded from Genbank (accessed 15/02/2022). When available, collection country, host and date were annotated (Supplementary data). All sequences were aligned using MAFFT (Standley 2013), recombination occurrence was evaluated using RDP4 (Martin et al. 2015) and GARD (Kosakovsky Pond et al. 2006) and a Maximum Likelihood phylogenetic tree was reconstructed using IQ-Tree (Nguyen et al. 2015), selecting as the best substitution model the one with the lowest Bayesian Information Criteria (BIC), calculated with the same software.

## Results

### Infection frequency

Twenty-one out of 92 samples (22.83%; 95CI: 14.72–32.75%) were obtained from diseased animals. Overall, 10 samples tested positive (10.87%; 95CI: 4.51–17.23%). Of those, 3 were collected from apparently healthy subjects (2 domestic and 1 stray dog) while the remaining from dogs with diarrhoea (*n* = 3) or bloody diarrhoea (*n* = 4). The Cq was lower in clinically diseased subjects (mean = 26.78; sd = 5.1) compared to healthy ones (mean = 35.41; sd = 1.41). Five complete VP2 sequences were obtained, all originating from diseased subjects (Table 1).

**Table 1:** Summary of signalment and clinical data available for Ethiopian dogs whose CPV-2 strains could be sequenced. Main phenotypic features have also been reported

										Variable AA positions					
Sample Code	Location	Species	Status	Sex	Age	Sample Source	Clinical Sign	CPV-2 Cq	Acc.Number	5	267	297	370	426	
16D	Clinic	Dog	Domestic	F	Young	Fecal	Bloody Diarrhoea	28,23	OM937842	G	Y	A	R	E	
17D	Clinic	Dog	Domestic	M	Young	Fecal	Diarrhoea	21,67	OM937843	G	Y	A	R	E	
18A	Clinic	Dog	Domestic	M	Young	Fecal	Diarrhoea	20,96	OM937844	A	F	A	Q	N	
18B	Clinic	Dog	Domestic	M	Young	Fecal	Diarrhoea	23,29	OM937845	G	Y	A	R	E	
18C	Clinic	Dog	Domestic	M	Young	Fecal	Bloody Diarrhoea	28	OM937846	G	Y	A	R	E	

### Sequence analysis

**Fig. 1:**
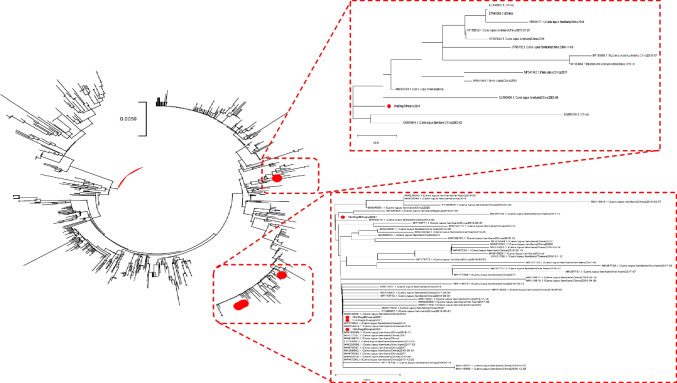
Maximum likelihood phylogenetic tree reconstructed based on the complete VP2 sequence of CPV-2 strains. In the right inserts, the clades including the CPV-2 strains sequenced in the present study (red circles) have been magnified. The collection host, country and year are reported for these sequences

All sequenced strains were classified as CPV-2. No recombination event was detected in the considered region.The mean genetic distance was 0.3% (maximum 1%) while the mean amino acid distance was 0.5% (maximum 1.1%). Different amino acids were observed at VP2 residues 5, 267, 370, 426 and 440 (Table 1). Based on amino acid 426, strain 16d, 17d, 18b and 18c were classified as CPV-2c while 18a as CPV-2 or CPV-2a. However, the analysis of other amino acid markers (i.e. amino acid 297) allowed its classification as new-CPV-2a (Decaro and Buonavoglia 2012). The phylogenetic analysis, based on genetic relationships, and thus depicting ancestry relationships, rather than on phenotype, confirmed such classification and the close clustering of 16d, 17d, 18b and 18c, as opposed to 18a. Regardless of the classification in antigenic variants, most of the strains with the closest genetic relationship with the Ethiopian ones were collected in China, although variants sampled in other Asian, but also African, countries were occasionally part of the same clusters (Fig. 1).

## Discussion

The present study describes, for the first time, the direct detection and genetic characterization of *Carnivore protoparvovirus 1* in Ethiopia, supporting previous serological evidences and complementing the knowledge of canine parvovirus circulation in Africa (Laurenson et al. 1998; Ogbu et al. 2020). As expected, being samples originating from dogs, all identified strains were CPV-2. Detection frequency, although not negligible, was significantly lower compared to what was reported in other African countries where CPV-2 presence was investigated, like Mozambique, Morocco, Nigeria, Tunisia, South Africa and Zambia (Touihri et al. 2009; Dogonyaro et al. 2013; Amrani et al. 2016; Figueiredo et al. 2017; Kapiya et al. 2019; Ndiana et al. 2021; Tion et al. 2021). However, it must be stressed that in those studies, only clinically diseased subjects were considered while a relevant percentage of apparently healthy dogs was included in the present one. If only dogs with enteric signs are considered, the infection frequency (i.e. 7 out of 21; 33%; 95CI: 14.59–56.97%) becomes comparable with the results of previous studies from other countries.

However, CPV-2 was detected also in healthy subjects, albeit at higher Cq, suggesting an asymptomatic infection or an incubation/convalescent carrier status. A potential vaccine strain detection can be confidently been excluded since no vaccination protocol is currently applied in Ethiopia. All tested stray dogs were negative, with only one exception represented by one, low-titer, asymptomatic subject, which could suggest a minor role of these populations in CPV-2 epidemiology. Nevertheless, the limited sample size of the present study and potentially unconsidered biases prevent any definitive conclusion. Thus, further and more extensive studies, should be performed to evaluate the epidemiological role of stray dog populations in Ethiopia.

Sequence analysis demonstrated the presence of two antigenic variants, CPV-2a and CPV-2c, with the latter being the most frequently identified. Such finding supports the heterogenicity of CPV-2 variants distribution in African countries since different regions and continents were demonstrated to host a different combination of antigenic variants, at a different frequency (Touihri et al. 2009; Dogonyaro et al. 2013; Amrani et al. 2016; Figueiredo et al. 2017; Kapiya et al. 2019; Ogbu et al. 2020; Ndiana et al. 2021; Tion et al. 2021). The reason behind this pattern is hard to be identified. Nevertheless, political, historical and economic reasons have shaped the relationship of different African countries with foreign ones that, in turn, display their peculiar molecular epidemiology (Miranda and Thompson 2016; Figueiredo et al. 2017; Tucciarone et al. 2018; Kapiya et al. 2019).

As described in Nigeria (Ndiana et al. 2021), the CPV-2a strain displayed the Ala-297 substitution, typical of viruses that have been designated as ‘‘new CPV-2a’’ (Decaro and Buonavoglia 2012; Hu et al. 2020). The comparison with available CPV-2 sequences highlighted a close relationship of all considered strains with Chinese ones. A strong economic connection has been developing between Ethiopia and China, with several Chinese businessmen travelling between the two countries or moving to Ethiopia, sometimes along with their pets (Cabestan 2012; Cook et al. 2016). A consistent epidemiological link can thus be expected and explained. Nevertheless, because of the limited data availability, particularly from Africa, other sources of viral introduction and spreading patterns cannot be excluded. In fact, variants with a certain genetic relationship were also detected in other Asian countries but also in African ones and CPV-2 is well known to have experienced a remarkable spreading over time involving different migration paths (Tucciarone et al. 2018; Mira et al. 2019; Balboni et al. 2021). If these or other countries played a role in the viral introduction to Ethiopia or simply shared a common importation source will require further investigations (Tucciarone et al. 2018; Ogbu et al. 2020; Balboni et al. 2021). The complete genome sequencing, instead of the VP2 only, could also allow a better discrimination of the viral migration paths. The strain clustering has sometimes been reported to slightly differ between complete genome and VP2-based analysis, potentially due to intergenic recombination events or convergent evolution masking the actual phylogenetic relationships. Therefore, although VP2-based analysis can be considered a good approximation of viral history and migration, it more formally represents a reconstruction of the gene flow and evolution over time and should thus be critically interpreted.

Considering the clinical relevance of CPV-2 infection and the risk it poses to local domestic and wild carnivore populations, especially endangered ones, much stronger monitoring and surveillance activity on foreign incoming animals should be performed. More strict constraints on animal introduction, e.g. compulsory vaccination, should also be considered. While a clear association between antigenic variants and clinical and epidemiological relevance is still debated, some evidence suggests an association between strain genetic features and virulence (Franzo et al. 2019). Therefore, more intense monitoring of CPV-2 characterization from a molecular/antigenic perspective, as well as the comparison among different countries over time should be performed.

In this sense, further studies would be of interest to investigate the occurrence of CPV-2 infection in wild animals and their relationship with local and foreign strains.

## Supplementary Information

Below is the link to the electronic supplementary material.

Supplementary file1 (FAS 995 KB)

## Data Availability

The datasets generated and analysed during the current study are available in the GenBank repository (Acc. Numbers: OM937842-46).

## References

[CR1] Alexander KA, Appel MJ (1994). African wild dogs (Lycaon pictus) endangered by a canine distemper epizootic among domestic dogs near the Masai Mara National Reserve, Kenya. J Wildl Dis.

[CR2] Amrani N, Desario C, Kadiri A (2016). Molecular epidemiology of canine parvovirus in Morocco. Infect Genet Evol.

[CR3] Balboni A, Niculae M, di Vito S (2021). The detection of canine parvovirus type 2c of Asian origin in dogs in Romania evidenced its progressive worldwide diffusion. BMC Vet Res.

[CR4] Butler JRA, du Toit JT, Bingham J (2004). Free-ranging domestic dogs (Canis familiaris) as predators and prey in rural Zimbabwe: threats of competition and disease to large wild carnivores. Biol Conserv.

[CR5] Cabestan JP (2012) China and Ethiopia: Authoritarian affinities and economic cooperation. China Perspectives. Online http://journals.openedition.org/chinaperspectives/6041. 10.4000/chinaperspectives.6041.

[CR6] Cook S, Lu J, Tugendhat H, Alemu D (2016). Chinese Migrants in Africa: Facts and Fictions from the Agri-Food Sector in Ethiopia and Ghana. World Dev.

[CR7] Cotmore SF, Agbandje-McKenna M, Chiorini JA (2014). The family Parvoviridae. Arch Virol.

[CR8] Craft ME, Vial F, Miguel E (2017). Interactions between domestic and wild carnivores around the greater Serengeti ecosystem. Anim Conserv.

[CR9] Decaro N, Buonavoglia C (2012). Canine parvovirus–a review of epidemiological and diagnostic aspects, with emphasis on type 2c. Vet Microbiol.

[CR10] Dogonyaro BB, Bosman AM, Sibeko KP (2013). Genetic analysis of the VP2-encoding gene of canine parvovirus strains from Africa. Vet Microbiol.

[CR11] Figueiredo J, Miranda C, Souto R (2017). Genetic characterization of canine parvovirus type 2 subtypes in Maputo, Mozambique. Arch Microbiol.

[CR12] Franzo G, Tucciarone CM, Cecchinato M, Drigo M (2017). Canine parvovirus type 2 (CPV-2) and Feline panleukopenia virus (FPV) codon bias analysis reveals a progressive adaptation to the new niche after the host jump. Mol Phylogenet Evol.

[CR13] Franzo G, Tucciarone CM, Casagrande S (2019). Canine parvovirus (CPV) phylogeny is associated with disease severity. Sci Rep.

[CR14] Hoelzer K, Parrish CR (2010). The emergence of parvoviruses of carnivores. Vet Res.

[CR15] Hu W, Xu X, Liu Q (2020). Molecular Characterisation and Genetic Diversity of Canine Parvovirus Type 2 Prevalent in Central China. J Vet Res.

[CR16] Hueffer K, Parrish CR (2003). Parvovirus host range, cell tropism and evolution. Curr Opin Microbiol.

[CR17] Hughes J, Macdonald DW (2013). A review of the interactions between free-roaming domestic dogs and wildlife. Biol Conserv.

[CR18] Kalli I, Leontides LS, Mylonakis ME (2010). Factors affecting the occurrence, duration of hospitalization and final outcome in canine parvovirus infection. Res Vet Sci.

[CR19] Kapiya J, Nalubamba KS, Kaimoyo E (2019). First genetic detection and characterization of canine parvovirus from diarrheic dogs in Zambia. Arch Virol.

[CR20] Kosakovsky Pond SL, Posada D, Gravenor MB (2006). GARD: A genetic algorithm for recombination detection. Bioinformatics.

[CR21] Laurenson K, Sillero-Zubiri C, Thompson H (1998). Disease as a threat to endangered species: Ethiopian wolves, domestic dogs and canine pathogens. Anim Conserv.

[CR22] Martin DP, Murrell B, Golden M (2015). RDP4: Detection and analysis of recombination patterns in virus genomes. Virus Evol.

[CR23] Mira F, Purpari G, di Bella S (2019). Spreading of canine parvovirus type 2c mutants of Asian origin in southern Italy. Transbound Emerg Dis.

[CR24] Miranda C, Thompson G (2016). Canine parvovirus: The worldwide occurrence of antigenic variants. J Gen Virol.

[CR25] Ndiana LA, Odaibo GN, Olaleye DO (2021). Molecular characterization of canine parvovirus from domestic dogs in Nigeria: Introduction and spread of a CPV-2c mutant and replacement of older CPV-2a by the “new CPV-2a” strain. Virusdisease.

[CR26] Nguyen LT, Schmidt HA, von Haeseler A, Minh BQ (2015). IQ-TREE: A fast and effective stochastic algorithm for estimating maximum-likelihood phylogenies. Mol Biol Evol.

[CR27] Ogbu KI, Mira F, Purpari G (2020). Nearly full-length genome characterization of canine parvovirus strains circulating in Nigeria. Transbound Emerg Dis.

[CR28] Otto CM, Drobatz KJ, Soter C (1997). Endotoxemia and tumor necrosis factor activity in dogs with naturally occurring parvoviral enteritis. J Vet Intern Med.

[CR29] Parrish CR (2010). Structures and functions of parvovirus capsids and the process of cell infection. Curr Top Microbiol Immunol.

[CR30] Paul Reed A, Jones E, v, Miller TJ,  (1988). Nucleotide sequence and genome organization of canine parvovirus. J Virol.

[CR31] Prittie J (2004). Canine parvoviral enteritis: A review of diagnosis, management, and prevention. J Vet Emerg Crit Care.

[CR32] Schoeman JP, Goddard A, Leisewitz AL (2013). Biomarkers in canine parvovirus enteritis. N Z Vet J.

[CR33] Shackelton LA, Parrish CR, Truyen U, Holmes EC (2005). High rate of viral evolution associated with the emergence of carnivore parvovirus. Proc Natl Acad Sci U S A.

[CR34] Sillero-Zubiri C, King AA, Macdonald DW (1996). Rabies and mortality in Ethiopian wolves (*Canis simensis*). J Wildl Dis.

[CR35] Standley K (2013). MAFFT multiple sequence alignment software version 7: improvements in performance and usability. (outlines version 7). Mol Biol Evol.

[CR36] Steinel A, Parrish CR, Bloom ME, Truyen U (2001). Parvovirus infections in wild carnivores. J Wildl Dis.

[CR37] Tion MT, Shima FK, Ogbu KI (2021). Genetic diversity of canine parvovirus variants circulating in Nigeria. Infect Genet Evol.

[CR38] Touihri L, Bouzid I, Daoud R (2009). Molecular characterization of canine parvovirus-2 variants circulating in Tunisia. Virus Genes.

[CR39] Tucciarone CM, Franzo G, Mazzetto E (2018). Molecular insight into Italian canine parvovirus heterogeneity and comparison with the worldwide scenario. Infect Genet Evol.

[CR40] Tucciarone CM, Franzo G, Legnardi M (2021). Genetic insights into feline parvovirus: evaluation of viral evolutionary patterns and association between phylogeny and clinical variables. Viruses.

